# National Medical Union, Manchester

**Published:** 1913-11-15

**Authors:** 


### NATIONAL MEDICAL UNION, MANCHESTER.

An important Conference of Non-Panel Medical Associations will be held in London at the end of this month. The National Medical Union, with the assistance of several leading London societies, is responsible for convening this meeting. Invitations are being issued to all known non-panel bodies, and the chief object of the Conference is the promotion of the federation of non-panel organisations throughout the country. The promoters aim at making the meeting representative of all non-service practitioners, and secretaries of all such Associations, and all medical men who are not serving under the National Insurance Act, are earnestly requested to communicate with the Secretary of the Union, Mr. J. Webster Watts, 5 John Dalton Street, Manchester. Notice of the time of the meeting will be sent to the lay and medical press at an early date.

